# Mindful Moderation: Differential Relationships Between Psychological Distress and NfL, GFAP and *p*‐tau217 by Diagnostic Group

**DOI:** 10.1002/alz70856_106010

**Published:** 2026-01-08

**Authors:** Christa Dang, Matthew JY Kang, Dhamidhu Eratne, Dennis Velakoulis

**Affiliations:** ^1^ University of Melbourne, Parkville, VIC, Australia; ^2^ National Ageing Research Institute, Melbourne, VIC, Australia; ^3^ Neuropsychiatry Centre, The Royal Melbourne Hospital, Parkville, VIC, Australia

## Abstract

**Background:**

Symptoms of depression, anxiety and stress have been associated with biomarkers of Alzheimer's disease and neurodegeneration, as well as increased risk of poor mental health and dementia. Understanding how these relationships vary across clinical groups may provide insight into modifiable risk factors and potential intervention strategies for neurodegenerative disease.

**Method:**

Participants were from the MiND Study (*N* = 98; control=62, ND=18, PPD=18). Multiple regression analyses examined relationships between psychological distress (DASS‐21 total score) and blood‐based biomarkers (neurofilament light (NfL), glial fibrillary acidic protein (GFAP) and *p*‐tau217)), with diagnostic group as a moderator. Covariates included age, sex, and weight.

**Result:**

Psychological distress levels significantly differed across all diagnostic groups (all *p* <.013), with the highest levels in PPD and the lowest in controls.

When also controlling for psychological distress, NfL was significantly higher for ND compared to control (*p* = .005) and PPD (*p* <.001) but did not differ between control and PPD (*p* = .161). GFAP levels were not significantly different between groups (all *p* >.300). *p*‐tau217 was higher for ND compared to controls (*p* = .012) but not PPD (*p* = .078). No difference was observed between controls and PPD (*p* = .701).

Psychological distress was associated with higher NfL for controls (b=.006, *p* = .009) but not for ND (*p* = .320) or PPD (*p* = .626), indicating a moderating effect of diagnostic group (*p* = .012). Psychological distress was significantly associated with higher GFAP for controls (b=.005, *p* = .027) and PPD (b=.004, *p* = .016), but not for ND (*p* = .032). The interaction was non‐significant. Psychological distress did not predict *p*‐tau217 for any diagnostic group.

**Conclusion:**

Psychological distress being associated with higher NfL and GFAP for controls suggests that distress may have harmful effects on brain health even in the absence of neurodegenerative or psychiatric conditions, highlighting the importance of mental health as a modifiable risk factor for future neurodegeneration.

The observed association between psychological distress and GFAP for PPD is consistent with prior research, which links chronic stress and psychiatric conditions to increased neuroinflammation. In contrast, the lack of an association between psychological distress and *p*‐tau217 reinforces its role as an Alzheimer's Disease‐specific biomarker, independent of psychological distress. Longitudinal research is needed to determine whether distress‐related neurobiological changes contribute to future neurodegeneration.

